# Proposed nomenclature for microhaplotypes

**DOI:** 10.1186/s40246-016-0078-y

**Published:** 2016-06-17

**Authors:** Kenneth K. Kidd

**Affiliations:** Department of Genetics, Yale University School of Medicine, 333 Cedar Street, New Haven, CT 06520-8005 USA

## Abstract

Microhaplotypes are a new type of genetic marker in forensics and population genetics. A standardized nomenclature is desirable. A simple approach that does not require a central authority for approval is proposed. The nomenclature proposed follows the recommendation of the HUGO Gene Nomenclature Committee (http://www.genenames.org): “We strongly encourage naming families and groups of genes related by sequence and/or function using a “root” symbol. This is an efficient and informative way to name related genes, and already works well for a number of established gene families…” The proposal involves a simple root consisting of “mh” followed by the two-digit chromosome number and unique characters established by the authors in the initial publication. We suggest the unique symbol be an indication of the laboratory followed by characters unique to the chromosome and laboratory. For instance, the microhaplotype symbol mh01KK-001 refers to a locus on chromosome 1 published by the Kidd Lab (KK-) as their #001. Publication defines mh01KK-001 as comprised of four single nucleotide polymorphisms (SNPs), rs4648344, rs6663840, rs58111155, and rs6688969.

**Electronic supplementary material:**

The online version of this article (doi:10.1186/s40246-016-0078-y) contains supplementary material, which is available to authorized users.

## Proposal

A microhaplotype locus has been defined as consisting of two to five (or more) single nucleotide polymorphisms (SNPs) within the length of a DNA sequence read, arbitrarily set at about 200 to 300 bp. This length has been chosen to make the loci phase-known in an individual who is genotyped by current massively parallel sequencing (MPS) [1–3]. The alleles at the locus are defined as the haplotypes comprised, at the defining SNPs, of the specific alleles seen on chromosomes in the population. Microhaplotypes have been advocated as potentially very useful in forensics and population genetics [1–3]. This nomenclature proposal is the result of our own lab’s nomenclature problems with microhaplotypes and builds upon previous experience with early DNA polymorphism nomenclature as well as ongoing issues in maintaining ALFRED [4]. The proposal is not meant to be dictatorial but to inspire thought and discussion. Feedback is welcome, especially positive and constructive feedback. Negative feedback is also welcome, especially if an alternative system is proposed.

In presenting and discussing data in papers, it is simply too cumbersome to use the series of SNP symbols, usually rs numbers from dbSNP, in each mention of a microhaplotype (microhap) locus or its alleles. Standard procedure in the scientific literature would be to define a short symbol/acronym early in the paper and use that throughout, e.g., the use of *SNP* for single nucleotide polymorphism. In our publications, to date, we have used the nearby gene or our lab symbols for the locus while acknowledging that is not ideal [5]. Other laboratories are now searching for and publishing microhaplotype loci [6, 7]. As different labs and authors may refer to the same microhap locus with different short symbols, how to facilitate cross-referencing and incorporating data into a common database can become a problem.

In the early 1980s, the gene mapping community established an initial system to catalog and establish symbols for DNA polymorphisms (e.g., [8]) known as D numbers. D numbers consist of the letter D (for “DNA”), the chromosome number, the letter S (for “site” or “sequence”) and a centrally assigned sequential catalog number. While for individual SNPs the dbSNP rs numbers have superseded the D number symbols, those symbols persist for many short tandem repeat polymorphisms (STRPs), including many commonly used in forensics, such as D18S51 (the 51st site cataloged on chromosome 18) and D3S1358 (the 1358th site cataloged on chromosome 3). Based on that experience (for several years I was in charge of the central cataloging and assigning of D numbers using resources at the Yale Human Gene Mapping Library), an analogous system could be accepted and used by the genetics community for microhaps. Note that this is analogous to the nomenclature used for open reading frames that may be functional genes, e.g., “C14ORF43,” which was one of the ad hoc microhaplotype “names” in Kidd et al. [2]. The problem is that there is no central authority with the funding to assign official names. (We note that the correct previous symbol, C14orf43, has now been replaced by a gene name, ELMSAN1 [9].)

**Fig. 1:**
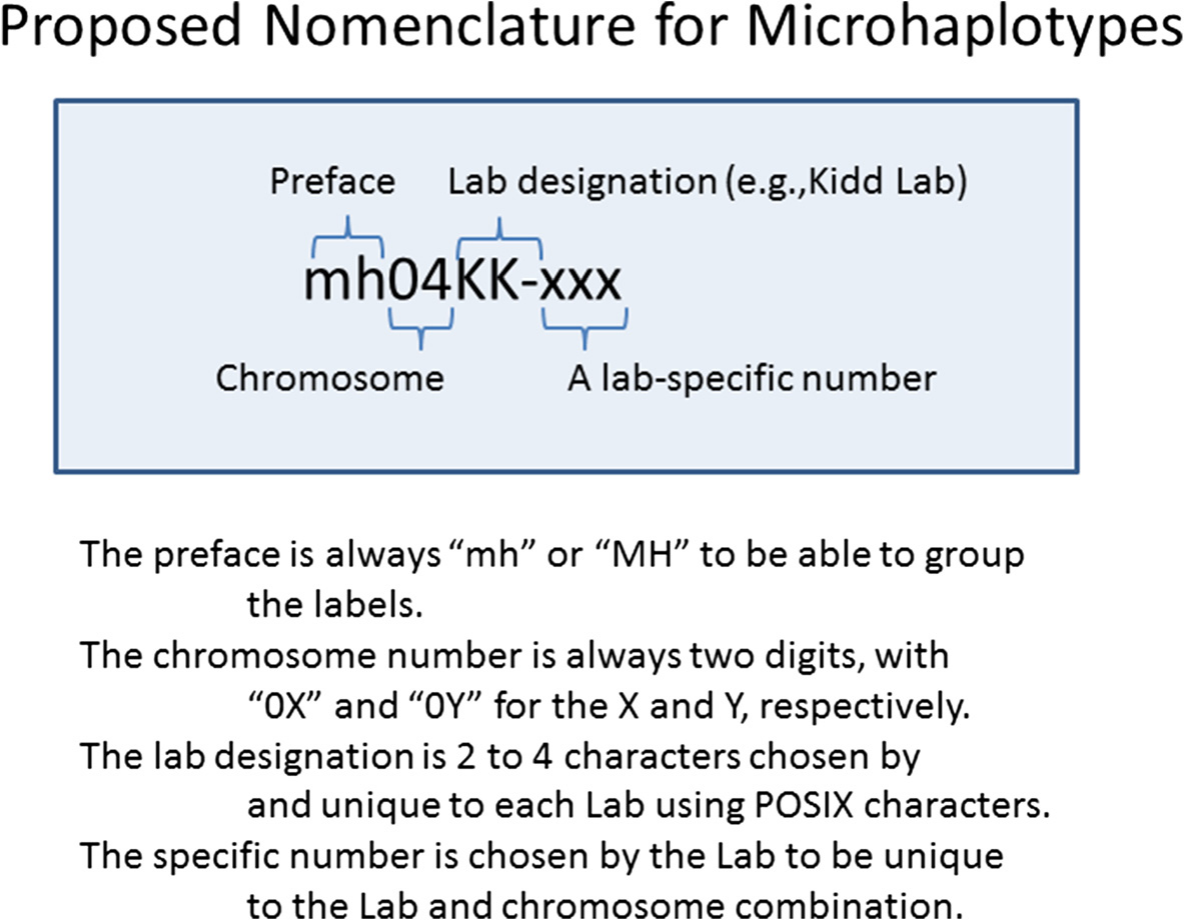
A graphic illustration of the nomenclature rules

**Fig. 2:**
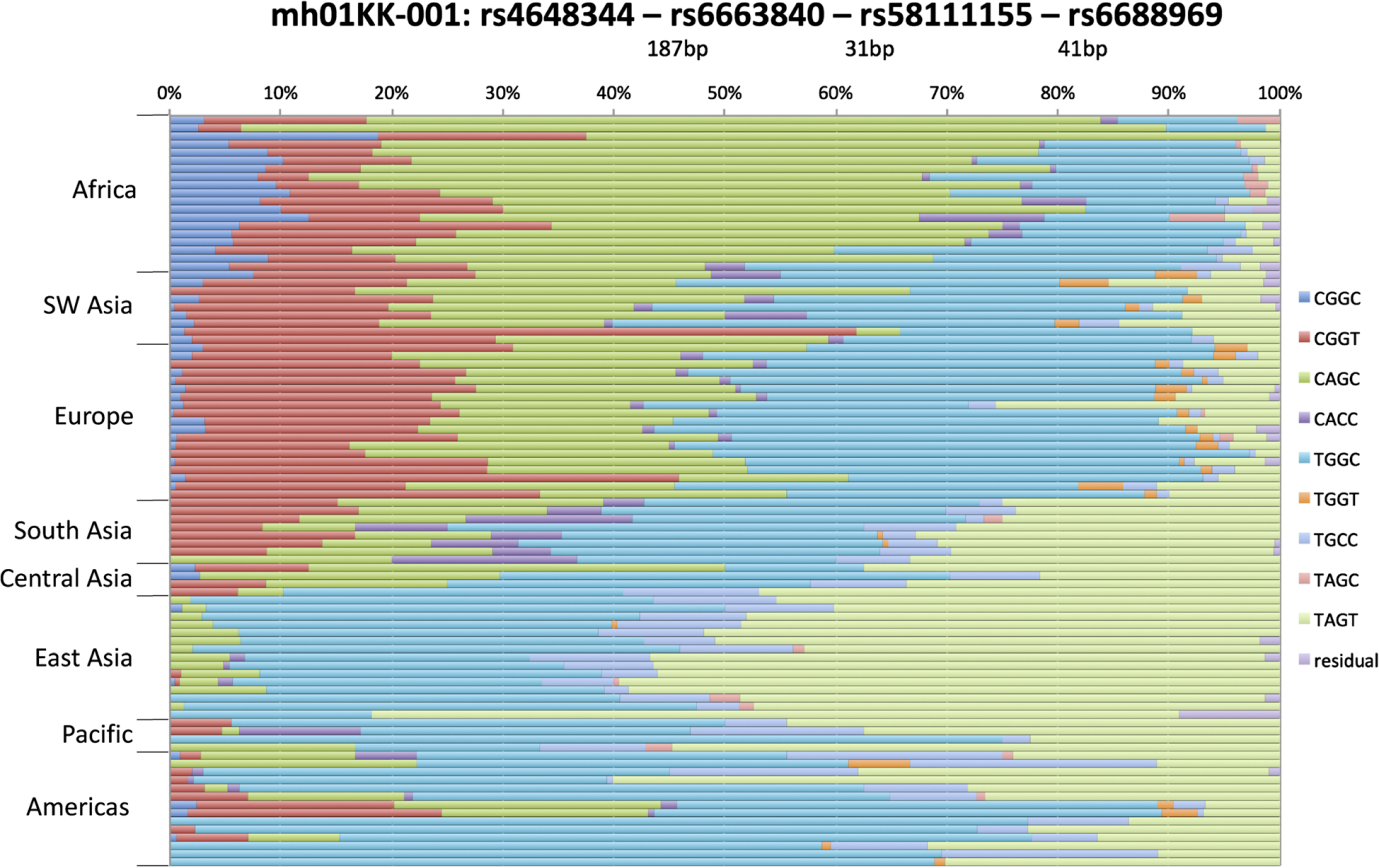
Population-specific allele frequencies. Each population is represented by a stacked bar representing the corresponding allele frequencies

A locus name should be compatible with nomenclature established by the HUGO Gene Nomenclature Committee [9]. Following the examples of D numbers and the orf nomenclature, we propose for discussion that the polymorphism symbol start with the letters “mh” (upper or lower case) followed by the chromosome number to distinguish the symbol from all current locus symbols and allow all microhap loci to sort together alphabetically and by chromosome number. This would eliminate most confusion with other locus symbols and provide immediately useful information. Rather than a centrally assigned sequence number, as was possible in the 1980s for D numbers, we propose the chromosome number be followed by a unique symbol of two to four characters as a symbol for the lab initially publishing the microhaplotype. This would then be followed by a unique catalog/sequence number established by that lab to be unique to the chromosome-laboratory combination. An example for the Kidd Laboratory involves an already published microhaplotype previously referred to as mh048 in Kidd and Speed [5] and previously as mh24:C14ORF43 in Kidd et al. [2]; this microhap becomes mh14KK-048, using “KK-” as the symbol chosen for Kidd Lab, and our lab assigned number “048” refers to the defining pair of SNPs, rs12717560 and rs12878166, on chromosome 14. The symbolism and its logic are illustrated in Fig. 1. Such symbols and their defining SNPs could easily be incorporated in a database, such as ALFRED [4, 10] with any previously published synonymous symbols. We are in the process of putting all of our microhaplotypes into ALFRED as an example of how such a system would work. Figure 2 illustrates population-specific allele frequencies for mh01KK-001, the microhaplotype locus noted above. The header defines the locus in terms of the SNPs involved; the nucleotides on the positive strand for those SNPs are used to define the alleles seen in the populations. Table 1 lists the proposed symbols for eight microhaplotypes included as figures illustrating allele frequencies in previous papers and abstracts [1, 2, 5]. Additional file 1: Table S1 lists the proposed symbols for the 31 microhaplotype loci in Kidd et al. [2]. Additional File 2: Figure S1 illustrates population-specific frequencies of four common haplotypes (and one rare one) found for a 282bp region downstream (pter) of the ADH7 gene.  The proposed nomenclature provides a “name” for this small and apparently non-functional intergenic region of no other particular interest.

**Table 1:** Examples of proposed symbolism for eight microhaplotypes previously illustrated in Kidd et al. 2013, 2014 [1, 2] and Kidd and Speed 2015 [5]

Symbol previously used	Standardized symbol	SNPs currently involved
EDAR	mh02KK-003	rs260694/rs11123719/rs11691107
RXRA	mh09KK-035	rs3118582/rs10776839
Microhap046	mh12KK-046	rs1503767/rs11068953
Microhap048	mh14KK-048	rs12717560/rs12878166
Microhap049	mh16KK-049	rs9937467/rs17670098/rs17670111/rs12929083/rs9926495
Microhap061	mh22KK-061	rs763040/rs5764924/rs763041
MicroTetrad180	mh11KK-180	rs12802112/rs28631755/rs7112918/rs4752777
MicroTetrad315	mh21KK-315	rs8126597/rs8131148/rs6517971

If subsequent papers would use the standardized symbolism proposed starting with the initial publication, considerable confusion could be avoided. Using this schema for naming microhaplotype loci, each lab could maintain its own records and create its own unique symbol when the lab’s microhaplotype data are published. The lab’s subsequent papers as well as papers by other researchers could use that as the symbol for that microhaplotype.

What we propose is not perfect; we recognize problems with definition of alleles or even the extent of the locus when additional variants are identified. Microhaplotype data obtained by MPS will include, in addition to the SNPs initially used to study the locus, other variations already known and characterized in dbSNP and 1000 Genomes, as other polymorphic sites or as rare single nucleotide variants (SNVs). Novel variation is likely to be identified when “new” populations are studied. In such cases, the initial SNPs specified become the initial basis for definition of alleles. While the same locus symbol would ideally be used, specification of the specific sites identified and definitions of alleles (haplotypes based on sites studied and identified) would be necessary in any publication. Possibly a system of indicating a modification of a previously defined microhaplotype could be devised rather than defining a completely new microhaplotype symbol. In the past, this has been the case with some studies of P450 genes (e.g., [11, 12]) because haplotypes were identified that did not correspond to the definitions in the “cypalleles” web site [13]. When individual SNPs are typed and haplotypes defined by statistical phasing, it is also possible that a SNP in the initial definition is omitted in a particular study. That could be specifically noted as the alleles are defined for that study. Manuscript-specific definition of alleles (haplotypes) will be less of a problem if at least a common symbol is used for the microhap locus more broadly defined.

Our own papers cited above illustrate the difficulty of maintaining a consistent symbolism when publications occur at different stages of the overall research in the lab. If subsequent papers used the standardized symbolism proposed starting with the initial publication, considerable confusion could be avoided. Each lab could maintain its own records and create its own unique symbol, but a common theme would preclude much potential confusion.

## Additional files

Additional file 1: Table S1. Proposed standardized symbols for the 31 microhaplotype loci in Kidd et al. [2]. The first column gives the symbol used in that paper. The second column lists the standardized microhaplotype locus symbol. The SNPs involved in these loci remain the same except for two. Microhaplotype mh01KK-001 has had two SNPs added (indicated by asterisks) extending the length to 259 bp. Microhaplotype mh01Nakahara (originally identified by Dr. Nakahara but not otherwise named by him) has one new SNP (also indicated by an asterisk) added by Kidd Lab; the new extent is 279 bp. (XLSX 11 kb) Additional file 2: Figure S1. Haplotype frequencies for a region between ADH7 and ADH1C. (XLSX 1.00 mb)
